# Catalytic Reduction of p-Nitrophenol on MnO_2_/Zeolite -13X Prepared with *Lawsonia inermis* Extract as a Stabilizing and Capping Agent

**DOI:** 10.3390/nano13040785

**Published:** 2023-02-20

**Authors:** Enshirah Da’na, Amel Taha, Mohamed R. El-Aassar

**Affiliations:** 1 Department of Biomedical Engineering, King Faisal University, P.O. Box 400, Alahsa 31982, Saudi Arabia; 2 Department of Chemistry, King Faisal University, P.O. Box 400, Alahsa 31982, Saudi Arabia; 3 Department of Chemistry, Faculty of Science and Technology, Al-Neelain University, Khartoum 1112, Sudan; 4 Department of Chemistry, College of Science, Jouf University, Sakaka 2014, Saudi Arabia

**Keywords:** zeolite, 13X molecular sieve, p-nitrophenol, p-aminophenol, catalytic reduction, manganese oxide, nanoparticles

## Abstract

p-nitrophenol (pNP) is a highly toxic organic compound and is considered carcinogenic and mutagenic. It is a very stable compound with high resistance to chemical or biological degradation. As a result, the elimination of this pollutant has been very challenging for many researchers. Catalytic reduction is one of the most promising techniques, if a suitable catalyst is developed. Thus, this work aims to prepare an eco-friendly catalyst via a simple and low-cost route and apply it for the conversion of the toxic p-nitrophenol (pNP) into a non-toxic p-aminophenol (pAP) that is widely used in industry. Manganese oxide was prepared in an environmentally friendly manner with the aid of *Lawsonia inermis* (henna) extract as a stabilizing and capping agent and loaded on the surface of 13X molecular sieve zeolite. The UV-Vis spectrum, EDS, and XRD patterns confirmed the formation of the pure MnO_2_ loaded on the zeolite crystalline network. The TGA analysis showed that the samples prepared by loading MnO_2_ on zeolite (Mn2Z, Mn3Z, and Mn4Z) lost more mass than pure MnO_2_ (Mn) or zeolite (Z), which is mainly moisture adsorbed on the surface. This indicates a better dispersion of MnO_2_ on the surface of zeolite compared to pure MnO_2_, and thus a higher number of active adsorption sites. SEM images and EDS confirmed the dispersion of the MnO_2_ on the surface of the zeolite. Results showed a very fast reduction rate, following the order Mn2Z > Mn3Z > Mn4Z > Mn > Z. With sample Mn2Z, 96% reduction of pNP was achieved in 9 min and 100% in 30 min. For Mn3Z, Mn4Z, and Mn, 98% reduction was achieved in 20 min and 100% in 30 min. Zeolite was the slowest, with only a 40% reduction in 30 min. Increasing the amount of zeolite in the synthesis mixture resulted in lower reduction efficiency. The kinetic study indicated that the reduction of p-nitrophenol on the surface of the prepared nanocomposite follows the pseudo-first-order model. The results show that the proposed nanocomposite is very effective and very promising to be commercially applied in water treatment, due to its low cost, simple synthesis procedure, and reusability.

## 1. Introduction

p-nitrophenol (pNP) is a highly toxic organic compound that is discharged to the water system from the pharmaceutical, photographic, fungicides, medicines, hair dyeing agents, leather, and corrosion inhibitors industries [1]. It causes many skin diseases and is considered carcinogenic and mutagenic [2]. Owing to the presence of the nitro group, pNP is a very stable compound with high resistance to chemical or biological oxidation [3]. Accordingly, many techniques such as adsorption [4], photocatalytic degradation [5], ozonation [6], membrane [7], microbial treatment [5], and electrocoagulation [8] have been applied for the removal of pNP from water. Among these processes, the conversion of the pNP into p-aminophenol (pAP) is very promising, because of its high efficiency, low cost, and simple operation [9,10]. Furthermore, when pNP is reduced, it forms pAP, which is non-toxic and widely used in pharmaceuticals [11,12], dyes, polymers, papers, explosives, corrosion inhibitors, and anti-corrosion lubricant [12,13,14].

Although microbial treatment of pNP provides an effective and safe method compared to the chemical treatment methods, it shows major shortcomings related to slow reaction rate and difficulty in finding suitable microorganisms [5]. In addition, it is vital to consider the cost, the feasibility of scaling up the process, environmental regulations, and operation requirements such as safety, maintenance, control, and robustness. Despite the feasibility of sodium borohydride (NaBH_4_) in reducing pNP, without a suitable catalyst, this reaction requires a high temperature and high hydrogen pressure [5]. Otherwise, this reduction is very slow [15]. Thus, it is crucial to find a suitable catalyst for this reaction to be able to take place under moderate conditions.

In the literature, a few researchers have reported the reduction of pNP over metal-free catalysts such as graphene and MOFs-based catalysts [12,16]. However, metal [17,18,19,20,21,22] and metal oxide [21,22,23,24,25] nanoparticles and nanocomposites have widely been applied, due to their excellent efficiency in lowering the reduction potential value [26,27,28]. Furthermore, their adaptable morphological, structural, chemical, physical, and optical properties have contributed to utilizing them in a wide area of research, including sensing, imaging, electronics, adsorption, and heterogeneous catalysis [15]. Additionally, the reduction of pNP using metal and metal oxide nanoparticle catalysts is very attractive, since it can be achieved in the aqueous solution [11]. Catalytic reduction of pNP to pAP with the aid of sodium borohydride (NABH_4_) on a nanoparticles catalyst has been investigated extensively in the literature with different types of nanoparticles or nanocomposite, such as AuCu/Pt nano alloy [15], silver nanoparticles [29], Au-ZrO_2_ nanocatalyst [30], Au [31], Pt, and Pd/SBA-15 [3], FeNi [27], Co_9_S_8_ [26], Pd/TiO_2_, [23], CuO, [24], magnetic Pt nanocomposites [32], Au-Pd [9], Au [33], SrTiO_3_/Ag [34], MoS_2_/ZnO [35], Ag/graphene [13], Au/TiO_2_ [25], PtPdBi, [36], Ag/ZnO/AC [37], Au/AC [2], Fe/chitosan [11], and Pd [17].

The catalytic performance of metals/oxide is strongly related to the particle size distribution and the dispersion of the nanoparticles on the surface of the support, if any exist [3]. Accordingly, one major requirement for a good nanoparticles-based catalyst is to minimize aggregation. Much effort has been dedicated to preparing hybrid catalysts by immobilizing nanoparticles on a surface of suitable support, such as activated carbon [2], silica-based materials [38], metal, chitosan [11], graphene [39], polymers [28], and zeolite [40]. Among all the reported supports, zeolites are considered very attractive supports for nanoparticles due to their high thermal stability, high surface area, nanoporous crystalline structure, stability in organic solvents, non-toxicity, eco-friendliness, availability at low cost, and corrosion resistance [40]. Accordingly, they have been used to support a wide range of nanoparticles for different applications [40,41,42,43,44].

The other strategy for minimizing aggregation is to surround the nanoparticles with a layer to protect aggregation. This layer could be polymeric materials, organic functionality, or a surfactant. Recently, a new approach was developed by utilizing plant extract as a stabilizing and capping agent [37,45,46,47,48,49,50,51]. The synthesis of nanoparticles with the aid of plant extracts offers a cheaper, safer, and eco-friendly pathway, since it removes the need for extra chemicals as reduction and capping agents. In addition, these plants are usually plentifully available at no or very low cost [31].

*Lawsonia inermis* (henna) belongs to the family Lythraceae and is a flowering plant, 2–6 m in height. *L. inermis* grows in North Africa, Asia, America, Australia, Egypt, and India [52]. The important natural constituents of *L. inermis* include tannins, phenolic compounds, alkaloids, and flavonoids, which are considered very effective reduction and capping agents [53].

MnO_2_ nanoparticles have been applied widely in catalysis, adsorption, oxidation reactions, dry cells, pigment, ceramics, electrodes, and batteries [43]. However, MnO_2_ has a major drawback related to its irregular pore structure that decreases the available surface area and, accordingly, the catalytic efficiency [40]. Furthermore, fine particles formed at relatively low temperatures tend to aggregate at high temperatures, leading to coarse crystalline materials with a particle size in the range of 2.5–10 μm. Similarly, the surface area decreases with an increase in the particle size distribution [54]. A promising solution for this shortcoming is to combine MnO_2_ with suitable support. In this regard, so far, a few studies have reported the synthesis of MnO_2_/zeolite composite. Thus, in this work, an effective and eco-friendly catalyst will be prepared by combining 13X zeolite molecular sieve as support with MnO_2_ nanoparticles prepared with the aid of *L. inermis* extract as a stabilizing and capping agent. The attractive characteristics of the proposed catalyst include the very simple and low-cost synthesis procedure, which eliminates the need for harsh chemicals such as NABH_4_ or critical conditions such as high temperature, the flow of inert gas, or multiple steps.

## 2. Materials

*Lawsonia inermis* (henna) powder was purchased from the local market of Al-Ahsa, Saudi Arabia. Zeolite (13X molecular sieves) (composition Na_2_O_3_, SiO_2_, H_2_O) was purchased from ThermoFisher, Waltham, MA, USA. MnCl_2_·4H_2_O (purity ≥ 99%), NaOH (purity ≥ 98%), and p-Nitrophenol (purity ≥ 99%) were supplied by Sigma-Aldrich (St. Louis, MO, USA) and used as received.

## 3. Method

### 3.1. Preparing Lawsonia inermis (Henna) Extract

To prepare the Henna extract (HE), 10 g of the plant root powder was mixed with 100 mL of distilled water. The mixture was boiled for 10 min then left overnight. After that, the mixture was filtered with Whatman filter paper and the filtrate was kept in the fridge until it was used.

### 3.2. Preparing the MnO_2_ Nanoparticles (M)

Henna extract (25mL) was mixed with 100 mL of 0.1 M MnCl_2_·4H_2_O solution. The pH of the mixture was adjusted to 11 using 1 M NaOH and an Orion 2-Star pH meter. The mixture was then stirred at room temperature and 70 rpm for 24 h. After that, the mixture was filtered and the solid was washed with distilled water and then ethanol. Finally, it was dried in a convection oven at 100 °C for 3 h, then collected and kept in a sealed container until it was used.

### 3.3. Preparing the MnO_2_ Nanoparticles Loaded on Zeolite

**Figure 1:**
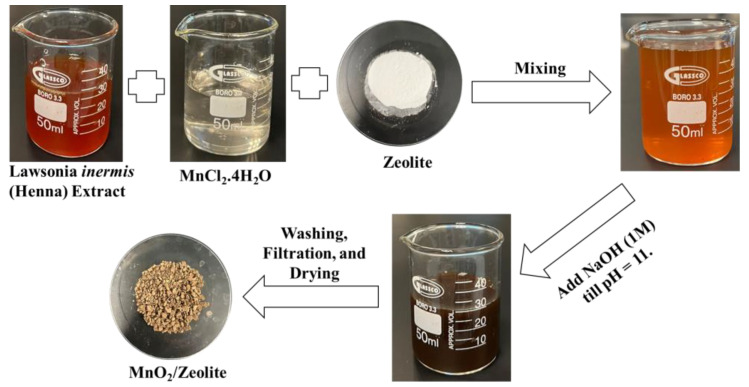
Schematic diagram of the synthesis of MnO_2_/Zeolite nanocomposite.

Figure 1 shows the steps followed in the synthesis of MnO_2_/Zeolite. Specifically, 100 mL of 0.1 M MnCl_2_·4H_2_O solution was mixed with 25 mL of the henna extract. A certain mass of zeolite (13X molecular sieves), according to Table 1, was added to the mixture and it was stirred for 30 min. The pH of the mixture was adjusted to 11 using 1 M NaOH and an Orion 2-Star pH meter. The mixture was then stirred at room temperature and 70 rpm for 24 h. After that, the mixture was filtered and the solid was washed with distilled water and then ethanol. Finally, it was dried in a convection oven at 100 °C for 3 h, then collected and kept in a sealed container until it was used.

## 4. Characterization

To confirm the formation of MnO_2_, all the nanocomposite samples were analyzed by X-ray diffraction (XRD) using XRD-7000 with a Cu-detector (Shimadzu, Tokyo, Japan) over the 2Ɵ range of 10–80°. Furthermore, they were dispersed in distilled water and the UV-Vis spectra were recorded for the wavelength range of 200–1100 nm using a UV-Vis spectrophotometer (Shimadzu, Tokyo, Japan). To test the thermal stability of the prepared samples, thermal gravimetric analysis (TGA) was conducted under nitrogen with a temperature ramp of 2°/min from 20 °C to 500 °C using a TGA-51 Shimadzu Thermogravimetric Analyzer (Tokyo, Japan). Dynamic light scattering (DLS) analysis was performed for the MnO_2_ nanoparticles to calculate the particle size distribution. This analysis was performed with a Thermo Scientific, Quattro S, USA. Scanning electron microgram (SEM) imaging was performed for all samples using a Thermo Scientific, Quattro S, USA. FTIR analysis was performed with a Thermo Scientific, Quattro S, USA for the wavenumber range of 4000–4000 cm^−1^. Nitrogen adsorption was measured for all samples after degassing the samples at 70 K, and the BET technique was performed to estimate the surface area. This analysis was performed with a NOVA 4200e (Quantachrome Instruments, B Beach, FL, USA).

### 4.1. Catalytic Reduction of p-Nitrophenol (pNP)

To study the catalytic activity of the prepared samples, 3 mL of 20 mgL^−1^ pNP solution was mixed with 1 mg of NaBH_4_ and 10 mg of Z, Mn2Z, Mn3Z, Mn4Z, or Mn was added to the solution; the concentration of pNP was monitored as a function of time by measuring the UV absorbance at 400 nm every 3 min for 30 min, using a UV-Vis spectrophotometer (Shimadzu, Tokyo, Japan). The removal percentage of pNP was calculated with Equation (1):

(1) $$R\%=\frac{A_{0}-A_{t}}{A_{0}}\times 100\%$$

where *A_t_* and *A*_0_ are the absorbance of p- nitrophenolate ion at any time t and at time zero, respectively. The kinetics of the catalytic reduction were tested with pseudo-first-order kinetics. The linear form of this kinetic model is given in Equation (2). The rate constants (k), were evaluated by plotting Ln (*A_t_*) versus time. The slope value of the straight line represents the rate constant k (min^−1^).

(2) $$Ln\;\left(A_{t}\right)=Ln\;\left(A_{0}\right)-kt\;$$

For the recycling experiment, the catalyst was collected by filtration, washed thoroughly with deionized water, and reused in the catalytic cycles.

### 4.2. FTIR Analysis

**Figure 2:**
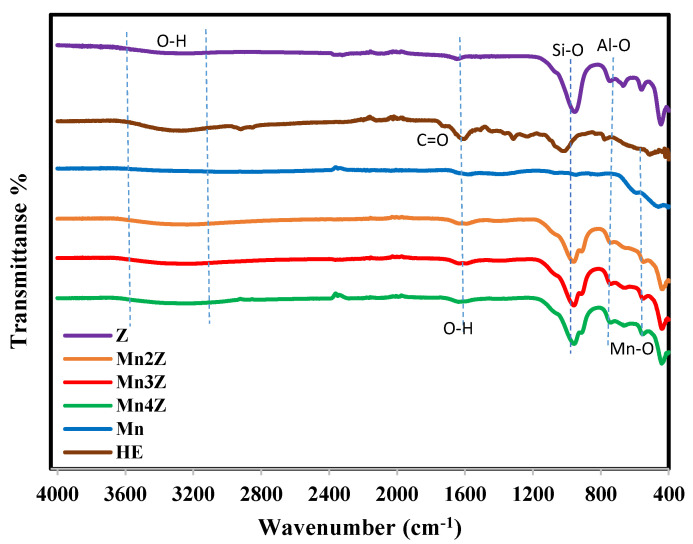
FTIR spectrum of, Z, Mn2Z, Mn3Z, Mn4Z, Mn, and HE for the wavenumber range 4000–400 cm^−1^. Spectra were shifted vertically for better visibility.

Figure 2 shows the FTIR spectra for Z, Mn2Z, Mn3Z, Mn4Z, Mn, and the plant extract (HE). All the patterns have a broad peak in the wavenumber range of ~3200–3600 cm^−1^, which is assigned to stretching vibrations of the O–H in hydrogen-bonded internal silanol groups and O–H stretching of water attached to the surface [41] The peak at ~1620 cm^−1^ is related to vibration bending of coordinated (–OH) groups attached to the zeolite surface. The peak at ~450 cm^−1^ in Mn2Z, Mn3Z, Mn4Z, and Mn is assigned to the Mn-O bond [14,42], while the peak at ~450 cm^−1^ in Z is related to the bending vibrations of Si–O and Al–O in zeolites [40]. The peak at ~740 cm^−1^ in Mn2Z, Mn3Z, Mn4Z, and Z is assigned to the stretching vibration of Al–O [55], while the peak at ~960 cm^−1^ in the same samples is related to Si–O stretching vibrations, and the asymmetric stretching of Si–O–Al tetrahedral [55]. In the HE pattern, the peak at ~1437 cm^−1^ is assigned to the C-O bond and that at ~1668–1725 cm^−1^ is related to the stretching vibration of the C=O cm^−1^ group of the effective compounds in the extract.

### 4.3. UV Analysis

**Figure 3:**
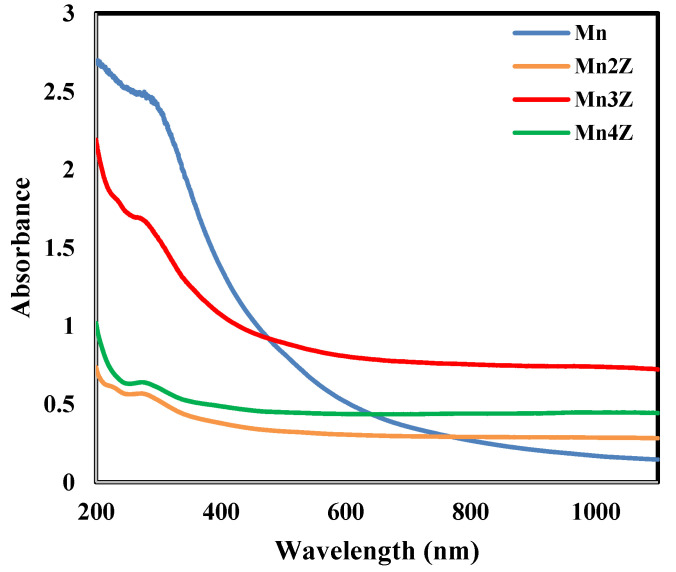
The UV-Vis spectra of Mn2Z, Mn3Z, Mn4Z, and Mn for the wavelength range of 200–1100 nm.

Figure 3 shows the UV-Vis spectra of Mn2Z, Mn3Z, Mn4Z, and Mn for the wavelength range of 200–1100 nm. The presence of the absorbance band in the range of 240–300 nm confirms the successful production of MnO nanoparticles. The shift of the absorption bands shows that different morphologies and sizes are presented [56].

### 4.4. TGA Analysis

**Figure 4:**
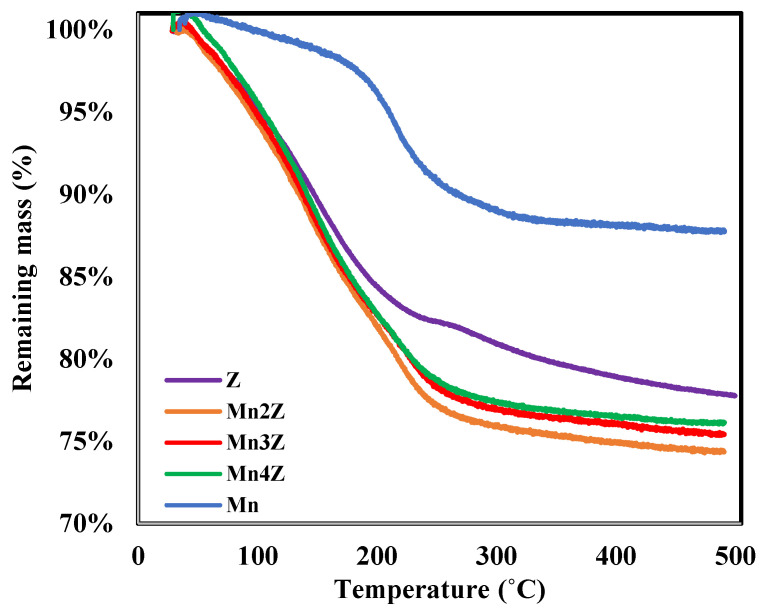
The TGA profile of, Z, Mn2Z, Mn3Z, Mn4Z, and Mn for the temperature range of 20–500 °C under nitrogen.

The TGA profile of the five samples is shown in Figure 4 for the temperature range of 20–500 °C. Pure MnO_2_ has the highest stability, with a mass loss of only 10%, which is mainly related to the water adsorbed on the surface. Bare zeolite lost 22%, mainly in the temperature range of 150–250 °C, which is also attributed to the water adsorbed on the surface. The three samples prepared by loading MnO_2_ on the surface of zeolite showed a higher mass loss (24–26%) within the same temperature range, indicating that the nanocomposite samples have higher adsorption capacity toward moisture. This indicates a better dispersion of MnO_2_ on the surface of zeolite compared to pure MnO_2_, and thus a higher number of active adsorption sites.

### 4.5. XRD Analysis

**Figure 5:**
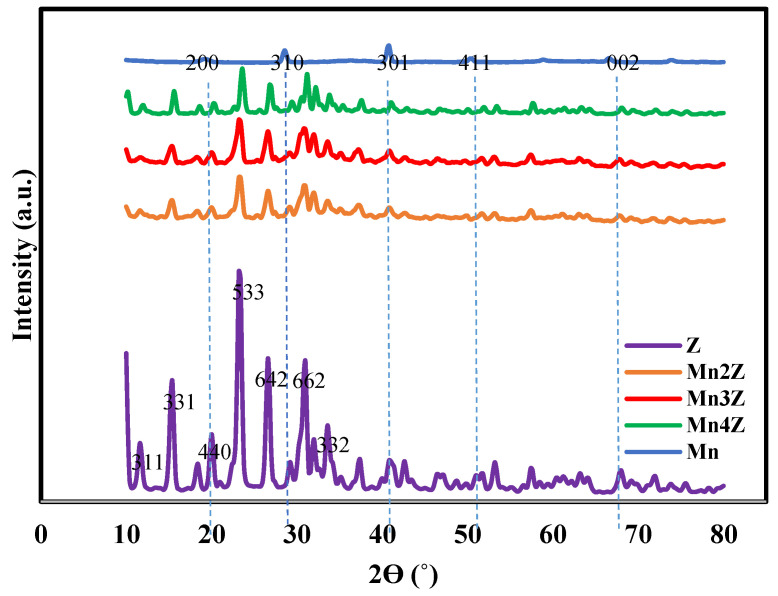
XRD patterns of, Z, Mn2Z, Mn3Z, Mn4Z, and Mn for the 2Ɵ range 10–80°. Spectra were shifted vertically for better visibility.

The phase purity and crystallinity of the prepared samples were investigated by X-ray diffraction technique (XRD), and the results are shown in Figure 5. The XRD patterns of Mn, Mn2Z, Mn3Z, and Mn4Z show peaks at 2θ values of 19.2, 28.6, 40.8, 50.4, and 66.6, which are attributed to (200), (310), (301), (411), and (002) crystal planes, respectively [57]. The presence of these diffraction peaks confirms the presence of the α-MnO_2_ crystal phase, according to the standard data (JCPDS card number 44-0141) [58]. Furthermore, extra peaks appear in the pattern of Mn2Z, Mn3Z, and Mn4Z at 2θ of 11.73, 15.2, 20.1, 23.3, 26.7, 31, and 33.8, corresponding to the planes of (311), (331), (440), (533), (642), (662), and (322), respectively, of the zeolite-13X substrate, which matches well with the pattern shown in sample Z related to pure zeolite [42,57]. These results confirmed the successful impregnation of MnO_2_ on the surface of zeolite-13X.

### 4.6. SEM and EDS Analysis

**Figure 6:**
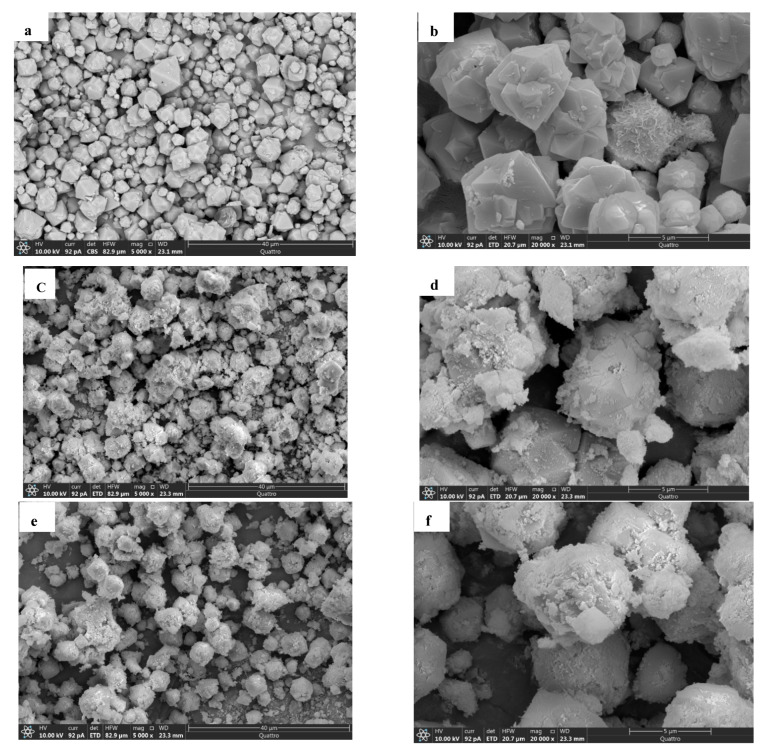

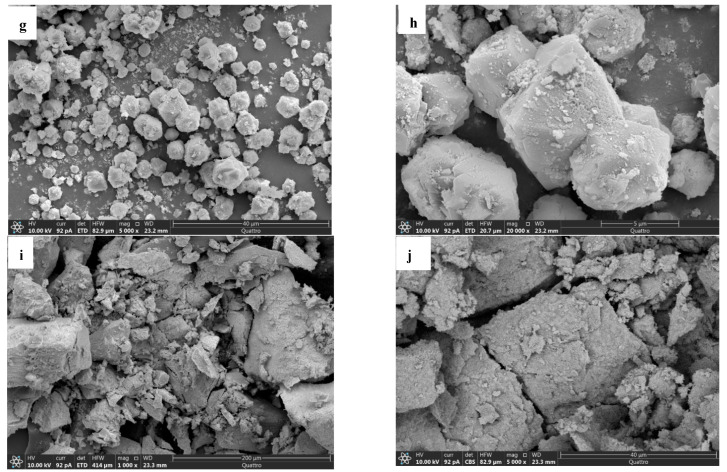
SEM images for Z (**a**,**b**), Mn2Z (**c**,**d**), Mn3Z (**e**,**f**), Mn4Z (**g**,**h**), and Mn (**i**,**j**) at two different magnifications.

**Figure 7:**
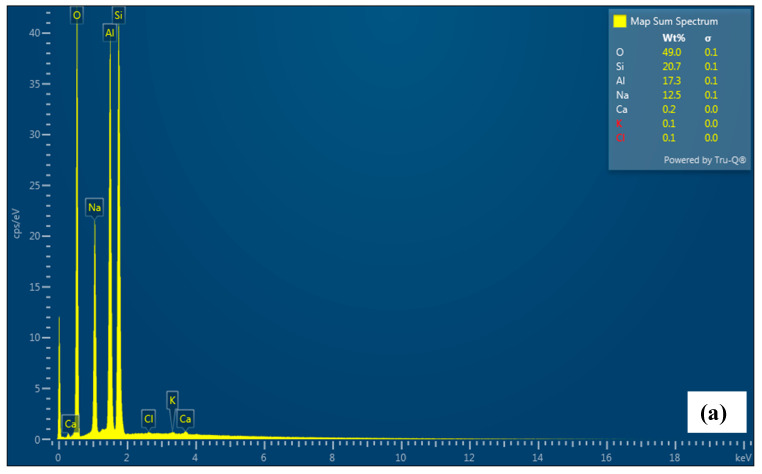

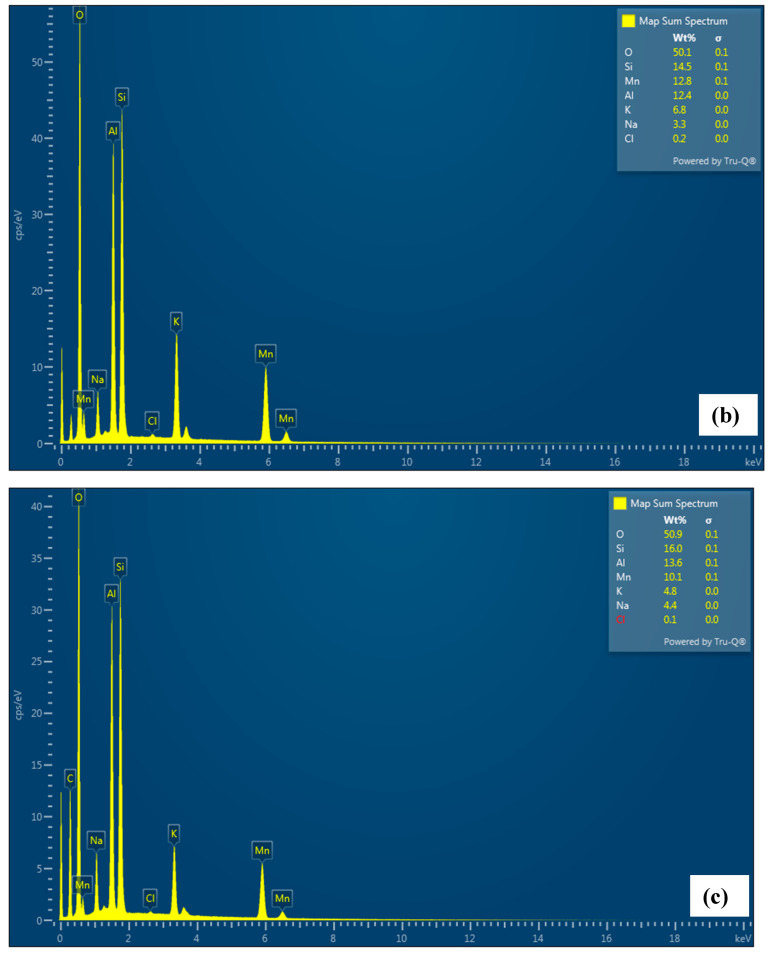

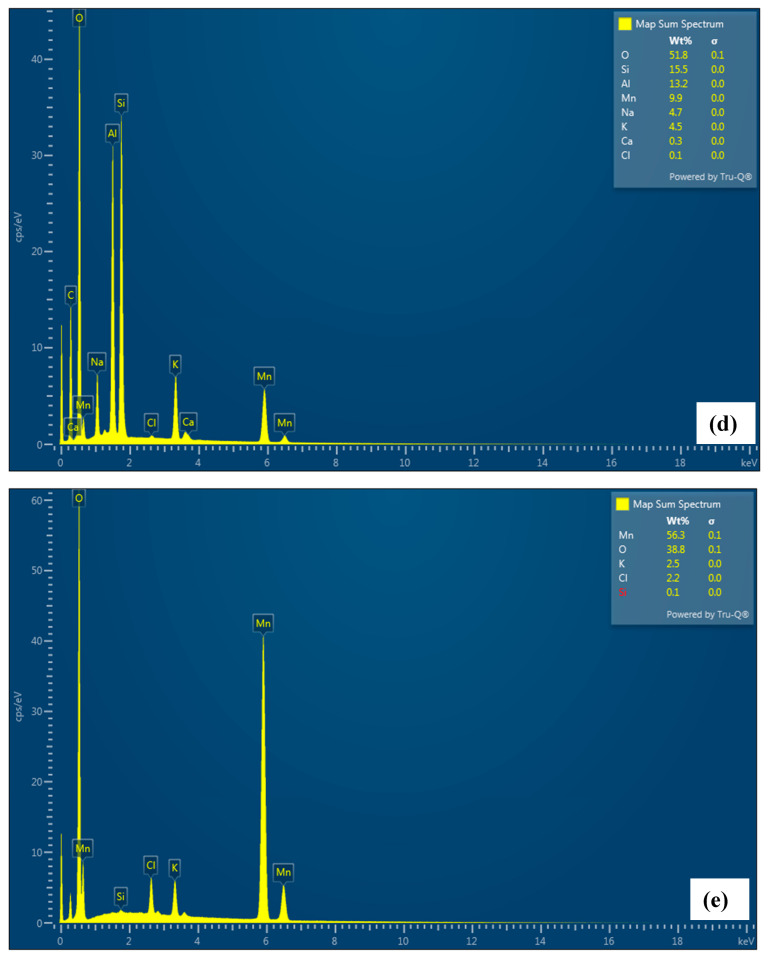
EDS analysis for Z (**a**), Mn2Z (**b**), Mn3Z (**c**), Mn4Z (**d**), and Mn (**e**).

The scanning electron micrograms of Mn, Mn2Z, Mn3Z, Mn4Z, and Z were taken at 5000× and 20,000× magnifications, as shown in Figure 6. The SEM data of bare zeolite show the typical crystalline structure, with smooth edges and crystallite sizes in the range of ~5–10 μm. [59] After depositing the MnO_2_ on the surface, the sharp edges are less clear for all samples. The images also indicate a good distribution of MnO_2_ on the surface of the zeolite-13X support and a low degree of MnO_2_ particle aggregation, with an average particle size of ~0.5 μm. Table 1 shows that the specific surface area, specific pore volume, and average pore diameter of pure zeolite, respectively, are 406.75 m^2^g^−1^, 0.722 cm^3^g^−1^, and 1.89 nm, indicating a microporous structure [60], while for the pure MnO_2_, the values are, respectively, 4.45 m^2^g^−1^, 0.0092 cm^3^g^−1^, and 3.17 nm. The EDS shown in Figure 7 confirms the dispersion of MnO_2_ on the surface of the zeolite. It is shown that the content of Mn decreased by increasing the ratio of zeolite in the sample, with Mn content of 56.3%, 12.8%, 10.1%, 9.9%, and 0.0% for Mn, Mn2Z, Mn3Z, Mn4Z, and Z, respectively.

**Figure 8:**
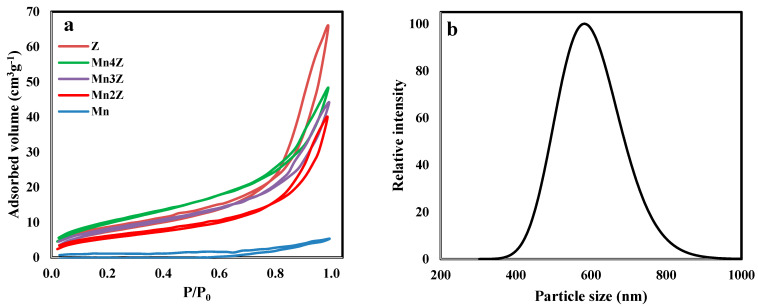
Nitrogen adsorption isotherms of Z, Mn2Z, Mn3Z, Mn4Z, and Mn (**a**), and DLS analysis of MnO_2_ (**b**).

The nitrogen adsorption/desorption isotherms are shown in Figure 8a. According to the IUPAC classification, the pure zeolite exhibits type II adsorption isotherm with a very slow increase in the adsorbed volume until a P/P_0_ of 0.8 is reached [60]. At this point, a sudden increase occurred due to the mesoporous structure of the zeolite sample. Furthermore, the appearance of the adsorption/desorption hysteresis loop is due to capillary condensation within the mesoporous structure. The isotherms for the samples Mn2Z, Mn3Z, and Mn4Z exhibit the same pattern, with a drastic decrease in the volume adsorbed, as shown in Table 1. This is mainly related to the blockage of the pores of zeolite by the accumulation of MnO_2_ particles. The isotherm of pure MnO_2_ displays a non-porous nature, with a very low pore volume, as shown in Table 1. Thus, the combination of MnO_2_ with the zeolite resulted in better dispersion of the nanoparticles, leading to a better catalytic activity, as will be discussed next. Combining MnO_2_ with the zeolite resulted in a drastic decrease in the surface area, pore volume, and average pore diameter for Mn2Z, Mn3Z, and Mn4Z. This is mainly due to the blocking of the pores of zeolite (1.89 nm) with the MnO_2_ particles, which have an average particle size distribution of around 600 nm, as shown in Figure 8b.

### 4.7. Catalytic Reduction of p-Nitrophenol (pNP)

**Figure 9:**
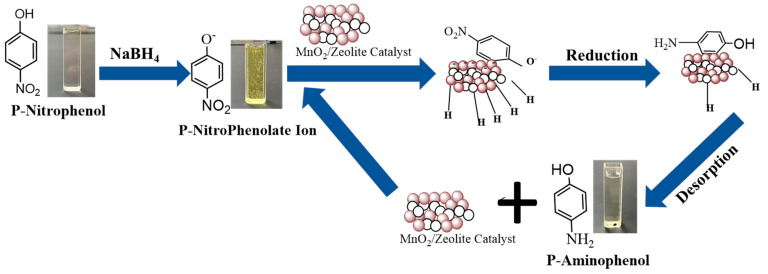
The mechanism of catalytic conversion of pNP into pAP.

**Figure 10:**
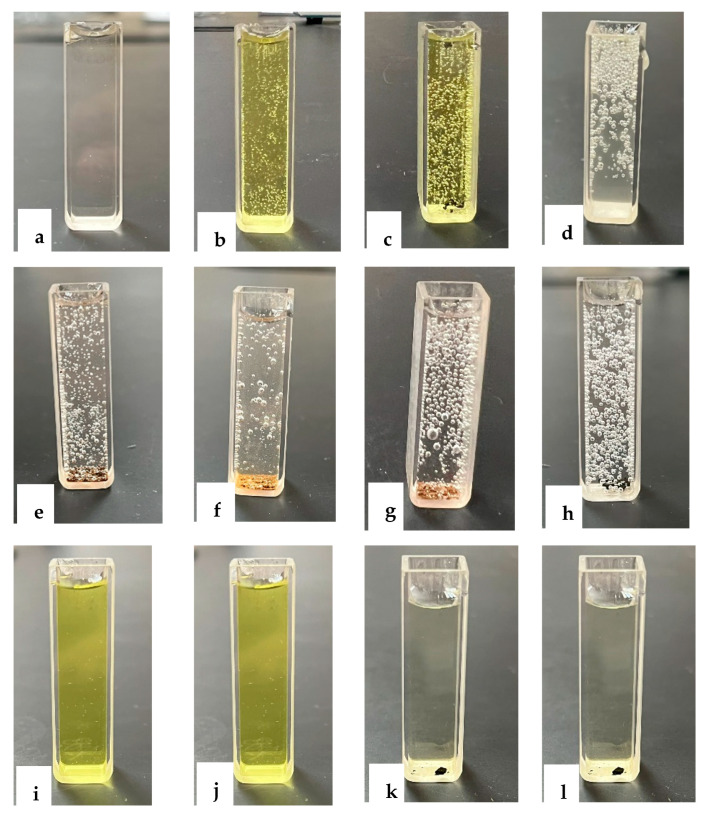
p-nitrophenol solution (20 mgL^−1^) (**a**), p-nitrophenol solution (20 mgL^−1^) directly after adding NaBH_4_ (**b**), p-nitrophenol solution (20 mgL^−1^) directly after adding NaBH_4_ and catalyst (**c**), p-nitrophenol solution (20 mgL^−1^) 30 min after adding NaBH_4_ and Z (**d**), Mn2Z (**e**), Mn3Z (**f**), Mn4Z (**g**), Mn(**h**), and without adding catalyst (**i**). p-nitrophenol solution (20 mgL^−1^) 30 min and 24 h after adding NaBH_4_ (**i**,**j**). p-nitrophenol solution (20 mgL^−1^) 30 min and 24 h after adding Mn2Z without NaBH_4_ (**k**,**l**).

**Figure 11:**
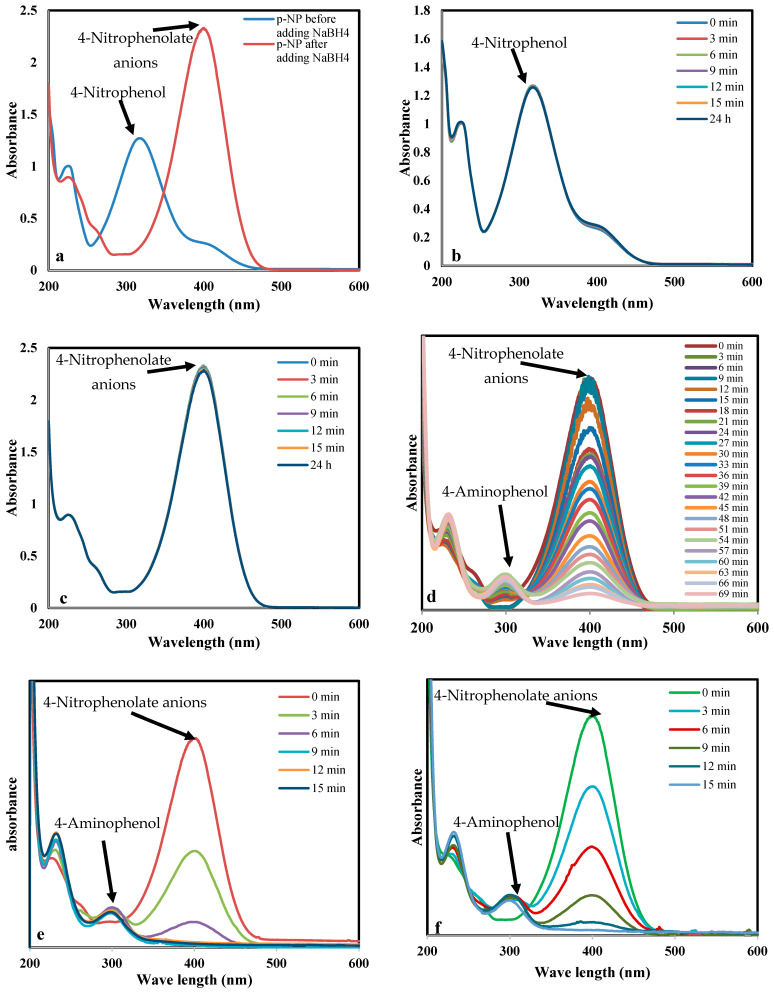

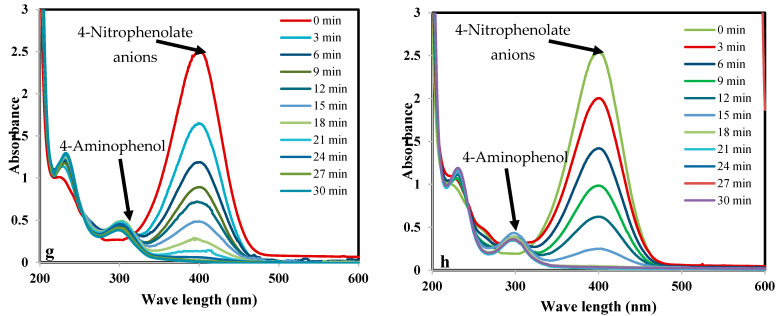
Time-dependent UV-Vis spectral changes of the pNP solution catalyzed by Z, Mn2Z, Mn3Z, Mn4Z, and Mn. The reduction conditions were: 3mL of 20 mgL^−1^ pNP solution, 1 mg NaBH_4_, and 10 mg of the catalyst.

The catalytic performance of the prepared nanocomposites was investigated using the reduction of p-nitrophenol into p-aminophenol according to the proposed mechanism shown in Figure 9. The characteristic absorption peak of pNP, which has a light yellow color as shown in Figure 10a, is located around 317 nm (Figure 11a). When NaBH_4_ is added to pNP, without a catalyst, the solution color changed to dark yellow immediately (Figure 10b). Furthermore, the peak position shifted from 317 to 400 nm (Figure 11a). These changes are related to the formation of 4-nitrophenolate anions, according to Equation (3) [5,61]. When the catalyst was added to the solution in the presence of NaBH_4_, the peak at 400 nm started to decrease, while a new peak around 300 nm started to appear and increase with time, due to the formation of pAP (Figure 11d–h). [2,5] Furthermore, the color of the solution started to disappear and it became colorless at the end of the reaction, as shown in Figure 10d–h, with the very large amount of gas bubbles confirming the reaction was taking place.

Two blank tests were performed to confirm the necessity of both catalyst and NaBH_4_. In the absence of NaBH_4_, adding the catalyst to the p-nitrophenol solution did not result in any reduction after 24 h (Figure 10k, Figure 8i and Figure 10b). This is mainly because the formation of p-nitrophenolate requires high pressure of hydrogen that is achieved by adding NaBH_4_ [5]. Furthermore, adding NaBH_4_ without the nanocomposites did not initiate the reduction into p-aminophenol, as observed from the solution color, which did not change after 24 h, as shown in Figure 10i,j. Furthermore, in the presence of nanocomposites without adding NaBH_4_ to the reaction mixture, there was no reduction or change in the absorbance peak (Figure 11c). Thus, for reduction to occur, both NaBH_4_ and nanocomposite are needed.

Figure 11 shows the time-dependent UV-Vis absorption spectra for the reduction of pNP with the aid of Z, Mn2Z, Mn3Z, Mn4Z, and Mn. The difference between Z, Mn2Z, Mn3Z, Mn4Z, and Mn is the catalytic activity and, thus, the time required to reach 100% reduction of pNP and the disappearance of the absorption peak at 400 nm. This peak decreased very slowly in sample Z, while Mn2Z showed the fastest rate of reduction. The high efficiency of Mn2Z may be related to the ratio of MnO_2_, which resulted in good loading of MnO_2_, with good distribution on the surface, leading to efficient accessibility of the reactants to the active sites and high catalytic performance.

**Figure 12:**
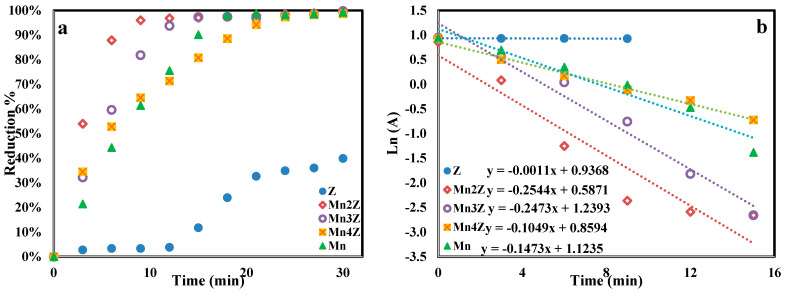
The efficiency of the catalytic reduction of pNP over Z, Mn2Z, Mn3Z, Mn4Z, and Mn nanocomposites (**a**), The linear plot of the pseudo-first-order kinetics reduction of pNP over Z, Mn2Z, Mn3Z, Mn4Z, and Mn nanocomposites (**b**). The reduction conditions were: 3 mL of 20 mgL^-1^ pNP solution, 1 mg NaBH_4_, and 10 mg of the catalyst.

Figure 12a shows the reduction percentage of pNP as a function of time for the five samples. It shows that Mn2Z is very fast, with a 96% reduction achieved in 9 min, compared to 82%, 65%, 61%, and 3% achieved by Mn3Z, Mn4Z, Mn, and Z, respectively. A complete reduction was observed by 30 min for all samples except the bare zeolite, with only a 40% reduction achieved. The performance of the nanocomposites prepared by combining the zeolite as support with the MnO_2_ (Mn2Z, Mn3Z, and Mn4Z) showed higher activity than Z and Mn. This is mainly related to improving the dispersion of MnO_2_ and thus minimizing the aggregation, resulting in a higher surface area and a higher number of active sites available for the reduction process. A pseudo-first-order kinetic model was used to fit the data, as shown in Figure 12b and Table 2. The results show that all the samples fit well with the pseudo-first-order kinetic model, with an R^2^ value of more than 0.90. The k values support the activity order of Mn2Z > Mn3Z > Mn ≅ Mn4Z >> Z.

**Figure 13:**
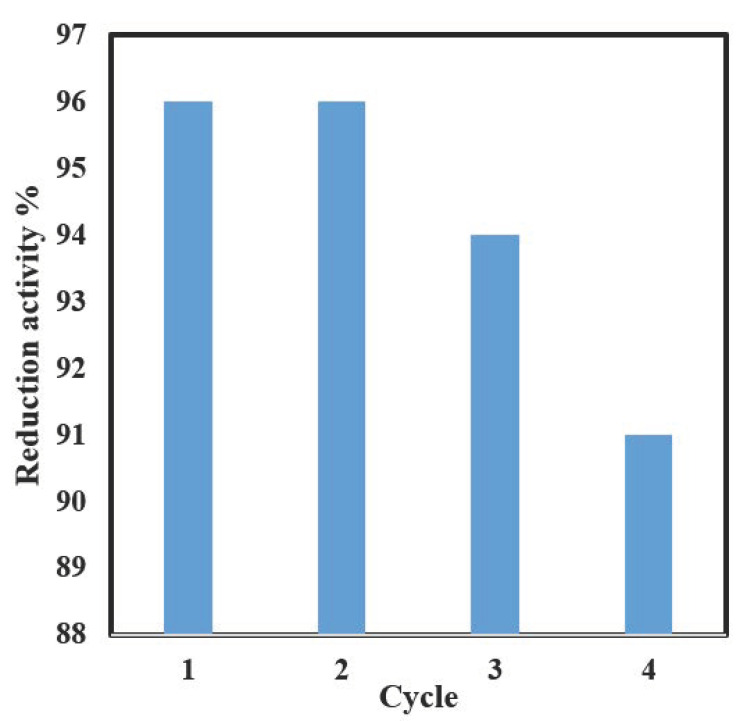
The efficiency of the catalytic reduction of pNP over Mn2Z. The reduction conditions were: 3 mL of 20 mgL^−1^ pNP solution, 1 mg NaBH_4_, and 10 mg of the catalyst for 10 min.

Table 3 shows a comparison of the reduction percentage obtained in this work with some other catalysts reported in the literature. As can be seen, the time required to complete the reduction in this study is comparable to most of the previously reported results. However, the catalyst reported in this work can be prepared from low-cost precursors and via very simple procedures, without the need for complicated conditions such as high temperature, multiple steps, a flow of inert gas, or the use of harmful chemicals such as NaBH_4_ or KBH_4_, as shown in Table 3. Furthermore, the developed catalyst showed very good reusability, by maintaining almost 95% of the original activity after four successive cycles, as shown in Figure 13.

## 5. Conclusions

Zeolite-supported MnO_2_ nanocomposite catalysts were synthesized successfully by utilizing plant extract as a stabilizing and capping agent. The synthesis route followed in this work was very simple, eco-friendly, cost-effective, and utilized very mild conditions. The catalytic efficiency of the nanocomposites was tested through the reduction of p-nitrophenol into p-aminophenol. Results indicated that almost 100% reduction was achieved in less than 30 min. Results of this study showed that a zeolite-supported MnO_2_ catalyst is very promising for the reduction of organic pollutants. Furthermore, the reusability of the catalyst was tested, and the results showed that 95% of the activity was maintained after four successive cycles, which is very important for commercial application.

## Figures and Tables

**Table 1:** Details of MnO_2_/Zeolite nanocomposite and N_2_ adsorption results.

Sample	Amount of Zeolite (g)	BET Surface Area (m^2^g^−1^)	PJH Pore Volume (cm^3^g^−1^)	PJH Pore Diameter (nm)
Mn	0	4.45	0.0092	3.17
Mn2Z	2	32.22	0.097	1.77
Mn3Z	3	29.45	0.131	1.56
Mn4Z	4	22.78	0.058	1.76
Z	Pure zeolite	406.75	0.722	1.89

**Table 2:** The fitting parameters of the linear plot of the pseudo-first-order kinetics reduction of pNP over Z, Mn2Z, Mn3Z, Mn4Z, and Mn nanocomposites.

	Z	Mn2Z	Mn3Z	Mn4Z	Mn
k (min^−1^)	0.0011	0.2544	0.2473	0.1049	0.1473
R^2^	0.9000	0.9094	0.9730	0.9912	0.9447

**Table 3:** Catalysts reported in the literature for the reduction of p-nitrophenol.

Catalyst	Operating Conditions	Degradation Efficiency	Ref.	Drawback
Ag/poly(norepinephrine)/MnO_2_	30 mL of 1 mM of 4-NP, 3 mL of 0.2 M of NaBH_4_, and After that, 1 mg of catalyst.		[62]	Multiple-step synthesis
AuNPs	2.5 mL of the 4-nitrophenol (8 × 10^−5^ M), 0.5 mL NaBH_4_ (0.6 M), and 0.25 mL gold nanocatalyst.	100% in 9 min.	[31]	Expensive precursors.
CuNPs	1.7 mL of p-nitrophenol (0.1 mM), 0.7 mL of NaBH_4_ (0.04 M), and an aqueous solution of Cu NCs (0.1 mL, 15 mM).	100% in 10 min.	[63]	
AuCu@Pt nanoalloys	100 mL of 10 nM AuNPs,100 mL of 1 mM 4-NP, 3.5 mL NaBH_4_ (100 mM).	100% in 10 min.	[15]	Complicated synthesis, expensive precursors.
AuNPs	2 mL of NaBH_4_ (0.1 M), 1 mL of 4-nitrophenol (2.0 × 10^−4^ M), and 2 μL of AuNP.	100% in 8 min.	[64]	Complicated synthesis, expensive precursors.
PtPdBi nanowire	3.3 mL of 0.09 mM p-nitrophenol and 0.10 mL of 0.10 M NaBH_4_ and 15 mg of the metal catalyst.	100% in 24 min.	[36]	High temperature, flow of argon.
AgNPs	4-NP (2 mL, 10^−4^ M), NaBH_4_ (1 mL, 10^−4^ M), and AgNPs (2.5 µL, 6 nmol).	96% in 3 min.	[20]	Use of NaBH_4_ during synthesis.
AuNPs	1.0 mL of 0.015 M NaBH_4_, 1.7 mL of 0.2 mM 4-nitrophenol, and 0.3 mL of the AuNPs colloidal suspension.	100% in 8 min.	[18]	Expensive precursors.
PdNPs	1 × 10^−4^ M of 4-NP (1.5 mL) and 5 × 10^−2^ MNaBH_4_ (1.0 mL, ice cold) 1 mgL^−1^ (0.5 mL) of PdNBs.	100% in 30 min.	[17]	Complicated synthesis, and flow of nitrogen.
Zerovalent iron NPs	4-NP (50 mgL^−1^) and 1.5% Pd/NZVI catalyst.	100% in 5 min.	[65]	Use of NaBH_4_ during synthesis.
Chitosan/CuNPs	100 μL of the colloidal catalyst, KBH_4_ solution. and 20 μL of 4.66 × 10^−2^ M p-NP.	100% in 30 min.	[66]	Use of KBH_4_ during synthesis.
Co_9_S_8_ nanotubes	1.0 × 10^−2^ M (4-nitrophenol), 2.0 × 10^−2^ M (NaBH_4_) and 10 mg L^−1^ (Co_9_S_8_ nanotubes).	100% in 8 min.	[26]	Complicated synthesis,
MOFs-derived N-doped carbon		100% in 10 min.	[12]	Complicated synthesis,
Carbon nanotube/Pd NPs	2 mL of 4-nitrophenol aqueous (5 × 10^−5^ M) and 1 mL of NaBH_4_ (0.05 M) and 100 mL of catalyst (0.05 gL^−1^)	100% in 7 min.	[67]	Complicated synthesis,
MnO_2_/Zeolite	3 mL of 20 mgL^−1^ pN, P 1 mg of NaBH_4_, and 10 mg of MnO_2_/Zeolite.	96% in 9 min.	This work	Simple synthesis

## Data Availability

Data available upon request.
